# Insights into the genetic landscape of cerebral palsy

**DOI:** 10.1002/ctm2.70412

**Published:** 2025-07-15

**Authors:** Yangong Wang, Changlian Zhu, Qinghe Xing

**Affiliations:** ^1^ Children's Hospital of Fudan University and Institutes of Biomedical Sciences of Fudan University Shanghai China; ^2^ Shanghai Center for Women and Children's Health Shanghai China; ^3^ Henan Key Laboratory of Child Brain Injury and Henan Pediatric Clinical Research Center Department of Pediatrics The Third Affiliated Hospital and Institute of Neuroscience of Zhengzhou University Zhengzhou China; ^4^ Center for Brain Repair and Rehabilitation Institute of Neuroscience and Physiology University of Gothenburg Gothenburg Sweden

1

Cerebral palsy (CP) is the leading cause of physical disability in children, characterized primarily by non‐progressive impairments in movement and posture.^1^ The estimated prevalence of CP ranges from 1.5% to 3.4% in live births.^2^ Although a number of factors, such as maternal infections, premature birth, or hypoxia before or during birth, as well as genetic factors, are known to increase the risk of CP, the aetiology of most cases remains unclear. The contribution of genetic variations to CP is increasingly recognized in the last 10 years.^3^ Different cohort selections have led to variations in the rate of genetic diagnosis, which is established in 10% to 50% of individuals with CP.^4^, ^5^ Simultaneous analysis of both single nucleotide variants (SNVs) and copy number variations (CNVs) from exome sequencing (ES) or genome sequencing (GS) has been performed on small CP cohorts, with exceptions such as meta‐analyses and a study by Moreno‐De‐Luca et al.^6^ To date, only about 3000 individuals with CP have been reported in ES and GS studies, which lag behind other neurodevelopmental disorders (NDDs). Large sample sizes are required to describe the full profile of genetic causes due to the high genetic heterogeneity of CP.^7^ Furthermore, population and ethnic biases need be addressed, as current studies mainly focus on cryptogenic CP and developed countries.^5^

To address the gap, we systematically examined the genetic landscape of 1578 children with CP ^8^ (as illustrated in Figure 1). By simultaneously analysing SNVs and CNVs, the overall diagnostic yield of the cohorts was 24.5% (387/1578). We identified 412 phenotype‐related SNVs across 219 genes in 329 patients, highlighting the presence of putative monogenic disorders unrelated to exogenous brain damage. Additionally, we identified 54 clinically significant CNVs in 1536 children with CP using ES data, which would have added 3.7% to the diagnostic yield of ES. The diagnostic yield of CNVs with ES is lower compared with CNV‐seq or CMA studies, due to the relative insensitivity of ES data for CNV analysis. Although CP is more common in male, with a male predisposition, sex‐stratified analysis did not detect any significant difference in the diagnostic rate between the sexes. Our hospital‐based Chinese cohort includes 505 girls and 1073 boys, selected without specific risk profiles, allowing for a comprehensive assessment of the frequency and diversity of genetic causes in CP.

**FIGURE 1:**
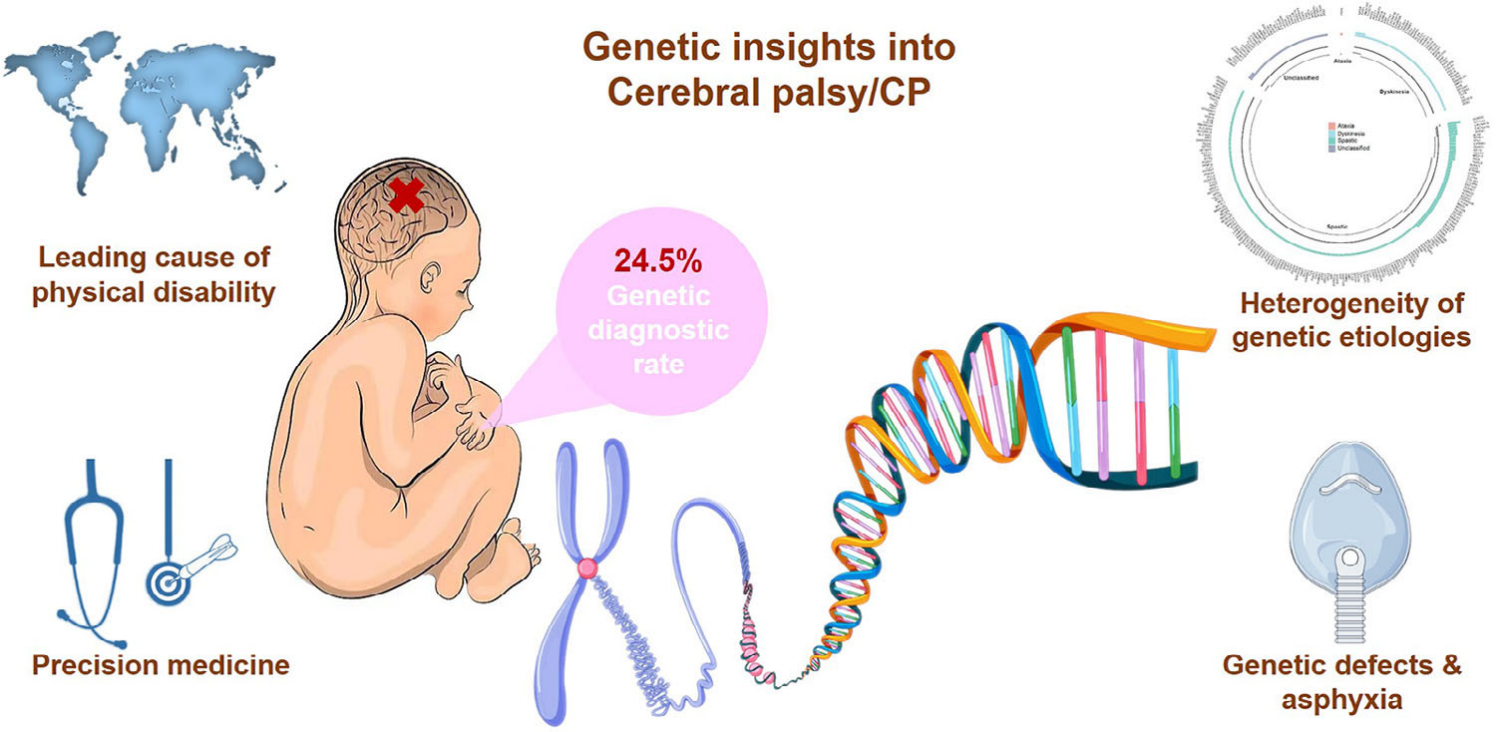
Genetic insights into cerebral palsy (CP). The figure illustrates the genetic insights into CP, emphasizing its genetic heterogeneity and the significance of genetic diagnostics. CP is the leading cause of physical disability in children globally. This study shows 24.5% of CP cases have a genetic diagnostic rate, highlighting the diverse genetic aetiologies that contribute to the condition. There is a link between genetic defects and asphyxia, which are significant factors in CP. Precision medicine and early genetic diagnosis are crucial for providing targeted interventions and improving clinical outcomes.

CP presented significant heterogeneity in genetic aetiology and clinical phenotype, posing diagnostic challenges.^9^ So far, no predominant gene was identified in CP cohorts. Among the 219 genes identified in our cohort, 66 genes had pathogenic (P) and likely pathogenic (LP) variants in 2 or more patients. The most frequently mutated gene was *KDM5C* detected in 6 individuals, followed by *ATP1A3*, *CACNA1G*, *CTNNB1*, *MECP2* and *TCF4* (5 cases each). In our cohort, some P/LP variants were characterized by incomplete penetrance or variable expressivity. For example, 2 out of 5 cases harboured P/LP variants in *CTNNB1* exhibited isolated motor disorders, while 3 others presented additional comorbidities.

CP is a spectrum of non‐progressive, permanent movement disorders defined by clinical description rather than aetiology.^10^ All progressive conditions should be excluded from CP according to this criterion. As genetic investigations continue, an increasing number of metabolic and genetic disorders is being recognized with the clinical manifestations of CP, sparking debate about whether these cases be included in CP.^11^ If some patients with variants in genes previously associated with progressive conditions present with CP phenotypes and are later diagnosed through genomic sequencing, these cases should remain described as CP and subtyped by genetic aetiology. It is challenging to identify genetic disorders in the CP cohort based on clinical manifestations alone. The current consensus is that findings from metabolic screens and genetic aetiology should not change the original CP diagnosis.^11^ This approach aligns with the stance for other NDDs, such as epilepsy,^12^ where the discovery of genomic causes hasn't altered the diagnosis. Excluding these cases could falsely reduce their perceived prevalence in the CP population in real‐world clinical practice, and including stable atypical cases of known genetic progressive disorders in CP diagnoses can enhance our understanding of their frequency in the CP population and the aetiology of CP itself.

Currently, we are still endeavouring to find effective prevention and treatment strategies for CP. As there are various genetic causes of CP, the strategies for successful interventions and management will also vary according to different genetic aetiologies. Among patients with positive diagnostic results in our study, 8.5% harboured clinically actionable genetic variants, enabling precision medicine to improve their outcomes. The proportion of cases with clinically actionable findings in other CP cohorts ranges from 8% to 30%.^13^ Early genetic testing of CP can bring about a paradigm shift in clinical practice.

Previous research indicated that cryptogenic CP shows a higher diagnostic yield compared with non‐cryptogenic CP, including findings from meta‐analysis.^5^ However, previous publications focused on the collective effects of known risk factors without analysing a single risk factor's impact on the diagnostic rate. The pathogenesis in CP cases varies based on exposure to different risk factors.^14^ Diagnostic yield specifically correlates with CP risk factors, for example, CP cases with prematurity have a lower diagnostic yield, while those with perinatal asphyxia exhibit a higher diagnostic yield than that of the overall cohort.^7^ Our findings imply that the CP and asphyxia are likely direct or secondary outcomes of inborn genetic defects in some CP cases with perinatal asphyxia.

Timely diagnosing a child with CP is challenging due to the variability of clinical features in young children. Delaying a clinical diagnosis can miss opportunities to improve clinical outcomes, as early interventions can optimize their impact on the developing brain's neuroplasticity. Genetic diagnoses can provide vast benefits, including early diagnosis and intervention, genetic counselling, avoiding unnecessary laboratory investigations and reducing legal risks. Genetic testing for CP is not routinely used in clinical practice, possibly due to the traditional view that genetic disorders are progressive, while CP is attributed to non‐progressive disturbances that occur in the developing brain. Genetic testing could direct efforts at multiple levels to improve the quality of life and prognosis for patients with CP and promote progress in CP research. Simultaneous analysis of CNVs and SNVs based on ES can provide a more comprehensive landscape of variations and lower cost compared with other strategies. Therefore, ES of young children with clinical manifestations of CP is highly recommended. Notably, our understanding of genetic variation contributing to CP phenotypes is still insufficient to guide clinical practice, and ES of additional large, unselected cohorts will be required to delineate the full range of genetic causes of CP.

## AUTHOR CONTRIBUTIONS

Yangong Wang, Changlian Zhu and Qinghe Xing wrote and edited the commentary.

## FUNDING INFORMATION

Shanghai Municipal Commission of Science and Technology Research Project, Grant/Award Numbers: 19JC1411000; the National Natural Science Foundation of China, Grant/Award Numbers: U21A20347; the Health Department of Henan Province, Grant/Award Numbers: SBGJ202301009; collaborative innovation center project construction for Shanghai Women and Children's Health.

## ETHICS STATEMENT

The authors declare no competing interests.
